# Factors associated with antidepressant responses to repetitive transcranial magnetic stimulation in antidepressant-resistant depression

**DOI:** 10.3389/fnins.2022.1046920

**Published:** 2022-12-02

**Authors:** Cheng-Ta Li, Tung-Ping Su, Chih-Ming Cheng, Mu-Hong Chen, Ya-Mei Bai, Shih-Jen Tsai

**Affiliations:** ^1^Department of Psychiatry, Taipei Veterans General Hospital, Taipei, Taiwan; ^2^Division of Psychiatry, School of Medicine, National Yang Ming Chiao Tung University, Taipei, Taiwan; ^3^Institute of Brain Science and Brain Research Center, School of Medicine, National Yang Ming Chiao Tung University, Taipei, Taiwan; ^4^Institute of Cognitive Neuroscience, National Central University, Zhongli, Taiwan

**Keywords:** treatment-resistant depression, Maudsley Staging Method, psychiatric admissions, repetitive transcranial magnetic stimulation, responses

## Abstract

**Background:**

A significant proportion of patients with major depressive disorder (MDD) failed to respond to antidepressant medications. Repetitive transcranial magnetic stimulation (rTMS) is an effective option for treating such treatment-resistant patients with MDD (TRD). Reliable clinical predictors for antidepressant responses to rTMS remain elusive.

**Methods:**

In total, 212 patients with MDD who failed to respond to at least one adequate antidepressant trial and had a detailed evaluation before rTMS were recruited for chart review. Demographic data, clinical characteristics, psychiatric comorbidities, symptom ratings [e.g., objective and subjective depression, life stress, depression refractoriness by Maudsley Staging Method (MSM)], and antidepressant treatment responses were analyzed.

**Results:**

MSM-subitem1 (duration of current depressive episode; Beta = 0.209, *p* = 0.004), MSM-subitem5 (a history of ECT treatment; Beta = –0.210, *p* = 0.004), and psychiatric admissions (Beta = 0.241, *p* = 0.001) predicted antidepressant response of rTMS treatment. ECT was underutilized (only 3.3%). Psychiatric admissions [Exp(B) = 1.382, *p* = 0.021], a comorbidity of OCD [0.047, 0.005], and life stress level [0.984, 0.029] predicted the history of ECT treatment.

**Conclusion:**

Several clinical variables (e.g., number of psychiatric admissions, OCD as a comorbidity, and life stress level) were reliable clinical factors associated with antidepressant responses of rTMS treatment and may be utilized in combination with MSM subitems to evaluate levels of TRD.

## Introduction

A considerable proportion of patients with major depressive disorder (MDD) failed to achieve significant or satisfactory improvements after undergoing multiple antidepressant treatments. Studies have reported that 33.3% of patients with MDD did not achieve symptomatic remission even after participating in sequential antidepressant trials (Rush et al., 2006, 2009). However, if the most commonly accepted definition of treatment-resistant depression (TRD; i.e., failure to respond to at least two adequate antidepressant trials) is applied, the prevalence of the aforementioned phenomenon can be as high as 44% (Rush et al., 2009).

In fact, TRD includes patients with a wide range of treatment refractoriness. Failure to respond to 1 adequate antidepressant trial had also been proposed as minimum requirement for TRD. For example, Fava and Davidson proposed that TRD patients can be defined as those who fail to respond to standard doses of at least 1 antidepressant administered continuously for at least 6 weeks (Fava and Davidson, 1996; Fava, 2003). Later, Thase and Rush proposed a staging model, which defines stage 1 of TRD as failure of at least 1 adequate trial of 1 antidepressant drug (Thase and Rush, 1997). On the other hand, high-frequency 10-Hz repetitive transcranial magnetic stimulation (rTMS) was approved by US FDA in 2008 for treating adult patients with antidepressant-resistant MDD, who have failed to achieve satisfactory improvement from prior antidepressant medication (i.e., 1 failed antidepressant trial) in their current episode (US Food and Drug Administration, 2008). The antidepressant efficacy of high-frequency rTMS has been demonstrated in meta-analytical studies, showing that the pooled response rates for rTMS were around 29.3% (Berlim et al., 2014) to 46.6% (Liu et al., 2014). In addition, theta-burst stimulation (TBS) is an updated form of rTMS that has more powerful and rapid effects on synaptic plasticity than traditional rTMS protocols (Huang et al., 2005). Randomized trials have indicated that intermittent TBS (iTBS) over left PFC has better antidepressant effects than sham treatment (Li et al., 2014, 2020) and is non-inferior to rTMS (Blumberger et al., 2018).

Compared with patients without TRD, patients with TRD have poorer clinical outcomes and incur higher healthcare costs (Li et al., 2012b; DiBernardo et al., 2018; Perez-Sola et al., 2021). For example, in our previous study, we examined data from a nationwide insurance database and discovered that patients with TRD had poorer psychiatric outcomes than those without TRD. These poorer outcomes included more psychiatric admissions and suicide attempts (Li et al., 2012a). The secondary analysis results of the Sequenced Treatment Alternatives to Relieve Depression study revealed that relative to patients without TRD (*n* = 2090), patients with TRD (*n* = 377) were slightly older [mean age of 44 years (patients with TRD) vs. 42 years (patients without TRD)] and had higher a baseline depression severity [Hamilton Depression Rating Scale (HDRS)-17 score of 24.4 (patients with TRD) vs. 22.0 (patients without TRD)] (DiBernardo et al., 2018). In addition, during their long-term follow-ups, patients with TRD were revealed to have a lower health-related quality of life in the mental and physical dimensions, more severe functional and work impairments, and productivity loss relative to patients without TRD (all differences were statistically significant) (DiBernardo et al., 2018). A register-based study conducted in Spain revealed that patients with TRD had a significantly higher odds ratio (OR) for death (OR = 1.92) and suicide-related events (OR = 1.30) than patients without TRD (Perez-Sola et al., 2021). Furthermore, suicide is a global public health problem with men dying at approximately twice the rate of women and depression accounting for approximately 50% of suicides (Li and Lee, 2022). Patients with TRD have a higher suicidal risk than patients without TRD; an increase in resistance (or refractoriness) increases the risk of suicide (Su et al., 2017; Perez-Sola et al., 2021; Reutfors et al., 2021; Li and Lee, 2022).

Patients with TRD include patients with MDD whose antidepressant refractoriness and resistance vary widely. Correctly measuring treatment resistance at the baseline can help clinicians to predict clinical outcomes and develop better treatment strategies. Several staging methods, such as the Thase and Rush method (Thase and Rush, 1997), the European Staging Method (ESM) (Souery et al., 1999), the Massachusetts General Hospital staging model (MGH) (Fava, 2003), the Antidepressant Treatment History Form-short form (ATHF-SF) (Sackeim et al., 2019), and the Maudsley Staging Method (MSM) (Fekadu et al., 2009b) have been proposed to quantify the treatment resistance levels of patients with MDD. The MSM model assigns a standard score for one or two failed antidepressant trials (i.e., Level 1: 1 point). Thus, it overlooks the most commonly accepted TRD definition, which is the failure to respond to at least two adequate antidepressant trials.

In contrast to most staging methods, which only account for failed trials involving antidepressant medications, augmentation or combination, or electroconvulsive therapy (ECT), the MSM also considers illness duration (current depressive episode) and symptom severity (Fekadu et al., 2009b). Illness duration is categorized into acute (≤12 months), subacute (13–24 months), and chronic (>24 months). Symptom severity is determined by the number of depression symptoms and the level of functional impairment (Fekadu et al., 2009b). Fekadu et al. (2009a) discovered that a higher MSM score at baseline significantly predicted functional impairment, persistent depression during a depressive episode, and the total number of months spent in depression. MSM scores that indicate mild and moderate TRD significantly predict more favorable responses to esketamine treatment (Lucchese et al., 2021).

However, a study that investigated the effectiveness of ECT reported that inpatients who were identified through the MSM to have TRD (*n* = 18) did not differ significantly from inpatients without TRD in terms of their depression scores at the time of psychiatric discharge (Ma et al., 2020). In addition, Hägg et al. (2020) compared patients with TRD who were identified through various staging methods, and they reported that MSM-identified TRD [adjusted hazard ration (aHR) = 0.95, 0.94–0.97] and MGH-identified TRD (aHR = 0.92, 0.92–0.94) were associated with a slightly reduced risk, whereas ESM-identified TRD was associated with higher non-significant, marginal risk for psychiatric hospitalization (aHR = 1.03, 95% confidence interval = 1.00–1.05). Thus, further investigations must be conducted to determine whether other clinical factors can enhance the predictability of the MSM for treatment resistance.

Moreover, although ECT is an effective option for TRD, it is severely underused in clinical practice in many countries, especially in Asia (Chanpattana et al., 2010). By contrast, high-frequency rTMS is an effective and commonly adopted treatment option for antidepressant-resistant MDD. However, not every patient responded to it. Therefore, in the present study a large clinical sample was reviewed to identify reliable clinical variables for predicting antidepressant responses to rTMS in MDD patients who failed to respond to at least one antidepressant medication.

## Methods

### Participants

Patients were eligible for the study if they were adults (aged between 21 and 70 years), were diagnosed with MDD on the basis of the criteria of the *Diagnostic and Statistical Manual of Mental Disorders, Fourth Edition*, and had failed to respond to at least one adequate antidepressant treatment during their current episode (e.g., failure to achieve a 50% improvement in depression after receiving an equivalent daily dose of 10 to 20 mg of escitalopram for at least 8 weeks). They were all recruited from a medical center mainly designed for treating MDD patients with inadequate responses to antidepressants (the Precision Depression Intervention Center) and had a detailed evaluation for clinical variables and symptom ratings before non-invasive brain stimulation. MDD diagnoses were established after a thorough medical history was determined and after the semi-structured Mini International Neuropsychiatric Interview (Sheehan et al., 1998) was conducted.

Patients were excluded if they had a lifetime psychiatric history of psychotic disorders, bipolar disorders, or organic mental disorder; had a lifetime medical history of major systemic illness or neurological disorder (e.g., stroke, seizure, traumatic brain injury, or post brain surgery); had brain implants (neurostimulators) or cardiac pacemakers; or were pregnant.

The authors assert that all procedures contributing to this work comply with the ethical standards of the relevant national and institutional committees on human experimentation and with the Helsinki Declaration of 1975, as revised in 2008. All procedures involving human subjects/patients were approved by local ethics review committee of Taipei Veterans General Hospital, with a waiver of informed consent, and the approval number was 2021-04-002BC.

### Study procedures and assessments

All recruited participants were carefully reviewed to obtain their demographic data (i.e., age, gender, marriage status, educational levels, occupation, and presence or absence of menopause) and verify clinical variables (duration of depression, past psychiatric admissions, suicide history, and psychiatric family history) and symptom ratings mentioned below.

The Clinical Global Impression–Severity (CGI-S) and HDRS-17 were used to objectively measure depressive symptoms (Hamilton, 1967), and the Depression and Somatic Symptoms Scale (DSSS) was used to subjectively measure depressive (DSSS-DS), somatic (DSSS-SS), and painful symptoms (DSSS-PS) (Hung et al., 2010). Life stress levels were assessed using a life event stress questionnaire (low, moderate, and high stress levels were defined by questionnaire scores of ≤149, 150–299, and ≥300, respectively) (Holmes and Rahe, 1967). Degree of treatment resistance or refractoriness was measured using the MSM (Fekadu et al., 2009a). The MSM measures five dimensions, namely duration of current depressive episode (MSM1), symptom severity (MSM2), failure of antidepressant trials (MSM3), use of augmentation (MSM4), and history of ECT treatment (MSM5) (Fekadu et al., 2009b).

Furthermore, the treatment response for non-invasive brain stimulation [e.g., 10-Hz repetitive transcranial magnetic stimulation (rTMS) and intermittent theta burst stimulation (iTBS) (Li et al., 2020)] for their current episode (the variable was labeled as “Responses to rTMS”) were recorded for all the participants. We used the Magstim Rapid^2^ stimulator (Magstim Co., Ltd., Wales United Kingdom) for the iTBS and rTMS protocols. The iTBS and rTMS parameters were the same as in our previously published work (Li et al., 2020). The parameters for iTBS protocol were three-pulse 50-Hz bursts administered every 200 ms, a 2-s train of bursts was repeated every 10 s, and 80% active motor threshold (MT), as measured from the right first dorsal interosseous muscle. One session of iTBS included a 2-s train of bursts repeated every 10 s for a total of 570 s (1800 pulses) to the left dorsolateral PFC (Li et al., 2020). The rTMS parameters were 10 Hz at 120% resting MT, with a stimulus train duration (on) of 4 s and an intertrain interval (off) of 26 s, for a total of 3000 pulses per session (Li et al., 2020). A total of at least 15 to 20 sessions of iTBS or rTMS over the left dorsolateral prefrontal cortex was regarded as an adequate course for a brain stimulation trial. Treatment response was defined as a 50% reduction from baseline in the HDRS-17 total score (Li et al., 2014).

### Statistical methods

All statistical analyses were performed using SPSS 21.0 (IBM, Armonk, NY, USA). Multivariable stepwise linear regression analyses were performed to identify the variables that were most predictive of responses to rTMS treatment. The rTMS treatment response was treated as the dependent variable. Independent factors were as follows: demographic variables (i.e., age, sex, marriage, education, job), clinical variables [i.e., duration of depression, psychiatric admission (times), past suicidal history, psychiatric family history, and menopause], psychiatric comorbidities (i.e., dysthymia, panic disorder, agoraphobia, social phobia, obsessive-compulsive disorder, post-traumatic stress disorder, alcoholic abuse, substance abuse, and generalized anxiety disorder), and symptom ratings (i.e., HDRS-17, DSSS, CGI, life stress, and MSM subitems). Multicollinearity was tested by calculating the variance inflation factor (VIF) score for each variable in the models, and the cut-off VIF score was set to 10 (Pokhrel et al., 2020). Likewise, a forward, stepwise logistic regression model was used to identify the optimal combination of predictors for MSM5 (ECT), with the history of ECT being treated as the dependent variable in the regression model. The optimal model with highest Nagelkerke’s R^2^ and the optimal predictors for Exp (B) and p value were identified. Correlations were verified through Pearson’s correlation test. Yates’s correction was used to compare the categorical variables (e.g., psychiatric admissions) of the examined groups. Statistical significance was set at p < 0.05 (two-sided tests).

## Results

In total, 212 patients with MDD who had at least one failed antidepressant trial were recruited for the present study (Table 1). The demographic data indicated that most of the participants were female (67.9%), had 12 to 16 years of education (61.3%), were unemployed (56.1%), and were not undergoing menopause (80.2%). For depression-related clinical variables, the participants’ mean [standard deviation (SD)] total duration of depression was 9.5 (8.8) years, and their mean number of past psychiatric admissions was 0.7 (2.3). Among the participants, 31.6% had a history of suicide attempts and approximately half (52.8%) had a psychiatric family history.

**TABLE 1:** Demographic data and clinical variables in the study population.

Variables	n (%)
Age in years, mean (SD)	42.4 (14.3)
**Female**/Male	**144 (67.9%)/**68 (32.1%)
**Unmarried**/Married/Divorced/Others	**101 (47.6%)/**92 (43.4%)/9 (4.2%)/10 (4.7%)
Education, ≤6/6–9/9–12/**12**–**16**/≥17yrs	11 (5.2%)/12 (5.7%)/38 (17.9%)/**130 (61.3%)/**21 (9.9%)
**Jobless**/Part-time/Full-time job	**119 (56.1%)/**18 (8.5%)/75 (35.4%)
Menopause, Yes/**No**/Male	42 (19.8%)/**103 (48.6%)/67 (31.6%)**
Duration of illness in years, mean (SD)	9.5 (8.8)
Past psychiatric admissions in times, mean (SD)	0.7 (2.3)
Past suicidal history, Yes/**No**	67 (31.6%)/**145 (68.4%)**
Psychiatric family history, Yes/**No**	100 (47.2%)/**112 (52.8%)**
MSM total score, mean (SD)	8.4 (2.1)
MSM1 (current episode): acute/subacute/**chronic**	87 (41%)/35 (16.5%)/**90 (42.5%)**
MSM2 (severity): subsyndromal/mild/**moderate**/severe/psychosis	3 (1.4%)/5 (2.4%)/**107 (50.5%)**/**91 (42.9%)/**6 (2.8%)
MSM3 (failed trials): 1–2/**3**–**4**/5–6/7–10/>10 antidepressants	68 (32.1%)/**70 (33%)/**37 (17.5%)/31 (14.6%)/6 (2.8%)
MSM4 (augmentation): **used**/not used	**142 (67%)/**70 (33%)
MSM5 (ECT): used/**not used**	7 (3.3%)/**205 (96.7%)**
**Psychiatric comorbidities**	
Dysthymia/Panic/Agoraphobia/Social phobia/	73 (34.4%)/40 (18.9%)/39 (18.4%)/25 (11.8%)/
OCD/PTSD/Alcohol abuse/Substance abuse/**GAD**	8 (3.8%)/3 (1.4%)/2 (0.9%)/1 (0.5%)/**158 (74.5%)**
Life stress, mean (SD)	147.3 (188.9)
Global impression: CGI-S, mean (SD)	4.6 (0.9)
Objective depression: HDRS-17, mean (SD)	22.4 (5.8)
Subjective depression: DSSS (DS/SS/PS), mean (SD)	20.5 (7.8)/11.9 (7.5)/6.0 (5.8)

SD, standard deviation; MSM, maudsley staging method; OCD, obsessive-compulsive disorder; PTSD, post-traumatic stress disorder; GAD, generalized anxiety disorder; CGI-S, clinical global impression-severity; HDRS-17, 17-item hamilton depression rating scale; DSSS, depression and somatic symptoms scale; DS, depressive symptoms; SS, somatic symptoms; PS, painful symptoms. Bold terms represent major results.

The patients’ mean (SD) total MSM score was 8.4 (2.1), which was a moderate to high level for treatment resistance. For the MSM subitems (Table 1), most of the participants had chronic episodes (42.5%), had moderate (50.5%) to severe (42.9%) depression, had failed to respond to three or four adequate antidepressant trials (33%), had undergone augmentation through non-antidepressants (68%), and had no history of ECT treatment (96.7%); all of these findings were within expectations. The Pearson’s correlation tests revealed that all the MSM subitems were significantly correlated with total MSM score (MSM1, *r* = –0.534; MSM2, *r* = 0.576; MSM3, *r* = 0.819; MSM4, *r* = 0.569; MSM5, *r* = 0.198; all *p* < 0.0001), but the correlation with the MSM5 (ECT treatment history) was the lowest.

For psychiatric comorbidities (Table 1), the highest prevalence was observed for generalized anxiety disorder (GAD; 74.5%), followed by dysthymic disorder (34.4%), panic disorder (18.9%), agoraphobia (18.4%), and social phobia (11.8%). A small proportion of the participants also had obsessive–compulsive disorder (OCD; 3.8%), post-traumatic stress disorder (PTSD; 1.4%), or alcohol or substance abuse (1.4%). Although the participants’ mean (SD) score for life stress was 147.3 (188.9), their clinical ratings indicated moderate to severe depression because they had a mean (SD) CGI-S score of 4.6 (0.9), mean HDRS-17 score of 22.4 (5.8), mean DSSS-DS score of 20.5 (7.8), mean DSSS-SS score of 11.9 (7.5), and mean DSSS-PS score of 6.0 (5.8).

### Significant predictors of antidepressant responses to repetitive transcranial magnetic stimulation treatment

**FIGURE 1:**
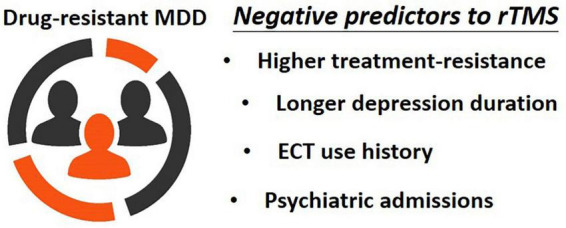
Factors associated with worse responses to rTMS in patients with antidepressant-resistant MDD. The illustration has been obtained from depositphotos.com.

The results indicated that the optimal model for predicting rTMS treatment outcomes by using all demographic data, clinical variables, psychiatric comorbidities, clinical ratings, and MSM subitems was statistically significant (*p* < 0.001). The model explained 10.2% (Nagelkerke’s adjusted R^2^) of the variance with only three factors (Table 2), namely MSM1 (duration of current depressive episode; Beta = 0.209, *T* = 2.933, *p* = 0.004), MSM5 (history of ECT treatment; Beta = –0.210, *T* = –2.907, *p* = 0.004), and psychiatric admissions (Beta = 0.241, *T* = 3.340, *p* = 0.001) (Figure 1).

**TABLE 2:** Step-wise regression models that best predicted the rTMS treatment responses and a history of ECT treatment.

Dependent	Independent variables	Adjusted R^2^	Significant predictors (*P*-value)	F-value (*P*-value)
rTMS treatment responses^f^	All^a^ + Comorbidities^b^ + Ratings^c^	0.102	MSM1-duration of current depression (0.004), MSM5- ECT treatment history (0.004), Psychiatric admissions (0.001)	8.182 (<0.001)
ECT treatment history^g^	All^a^ + Comorbidities^b^ + Ratings^e^	0.397	Psychiatric admissions (0.021), OCD ^d^ (0.005), Stress (0.029)	–3.372 (< 0.001)

^a^All variables include demographic variables (age, sex, marriage, education, job), clinical variables [duration of depression, psychiatric admission (times), past suicidal history, psychiatric family history, and menopause].

^b^Psychiatric comorbidities (by MINI).

^c^Symptom ratings: depression (HDRS-17, DSSS), clinical global impression-severity (CGI), and stress (life stress), subitems of Maudsley Staging Method for treatment refractoriness (MSM).

^d^OCD, a comorbidity of obsessive-compulsive disorder.

^e^All symptom ratings, except MSM5 (ECT history).

^f^Antidepressant failure only (1 point), Antidepressant failure + one kind of rTMS failure (2 points), Antidepressant failure + ≥2 different kinds of rTMS failures (3 points).

^g^By using forward, stepwise logistic regression, showing best model with highest Nagelkerke R^2^ and best predictors in the model [Exp(B)/*P*-value].

We further discovered that psychiatric admissions [Exp(B) = 1.382, *p* = 0.021], a comorbidity of OCD [Exp(B) = 0.047, *p* = 0.005], and life stress level [Exp(B) = 0.984, *p* = 0.029] predicted the history of ECT treatment (Table 2).

The aforementioned findings suggested the role of psychiatric admissions as an independent predictor for antidepressant treatment responses in patients with MDD. We further found that 26.4% (56/212) of the participants had at least one psychiatric admission in the past; specifically, 29 had one admission, 13 had two admissions, 6 had three admissions, 2 had four admissions, 2 had eight admissions, and 3 had ≥ 10 admissions. Furthermore, we discovered that the proportion of participants with three or more admissions (17.9%, 12/67) was significantly higher among the participants with a total MSM score of more than 10 than among the participants with a MSM score of 9 or less (0.7%, 1/145; *p* < 0.001). Similarly, a history of three or more admissions (9.4%, 13/138) was only identified among the patients with MDD who had a history of one or more rTMS treatment failures.

## Discussion

Using a large clinical sample, this study identified several reliable, clinical variables for predicting high treatment refractoriness of antidepressant-resistant MDD (e.g., poor responses to rTMS), which included current depression duration (MSM1), a history of ECT use (MSM5), more past psychiatry admissions, a comorbidity of OCD, and higher life stress. Reliable clinical predictors for rTMS or even TRD are important, since antidepressant options and parameters of rTMS treatment may be adapted according to the treated patients in clinical settings. The supporting evidence was that the combined use of MSM1 (duration), MSM5 (ECT), and number of psychiatric admissions allowed for the accurate prediction of rTMS treatment outcomes. In addition, we discovered that combining the number of past psychiatric admissions with a comorbidity of OCD and lift stress allowed for the reliable prediction of ECT use.

A previous review in 2012 had revealed that a high score of treatment resistance, a long duration of current episode, older age, and psychotic symptoms are negative predictors for treatment response to rTMS (Dumas et al., 2012). Results from a recent study had similar findings, which investigated clinical predictors of high-frequency rTMS for treating antidepressant-resistant bipolar and unipolar depression (*n* = 40) (Poleszczyk et al., 2018). They reported that longer duration of illness, higher number of prior hospitalizations, and more disturbed activity were associated with a worse response to rTMS (Poleszczyk et al., 2018). By increasing the sample size, we found that longer duration of illness and higher treatment resistance (i.e., MSM subitem 1 and 5), but not age and psychotic symptoms, were consistently associated with worse responses to rTMS.

We also found that the history of ECT treatment was rare (only 3.3% of the participants had a positive history of ECT use), which may compromise its sensitivity for predicting refractoriness in patients with MDD. Such a notion was supported by a European multicenter study involving 916 patients with TRD (defined as having two antidepressant trial failures); that study reported that inpatient status (OR = 1.65), a long duration of current episode (OR = 1.022), symptom severity (OR = 3.31), previous use of a high number of previous antidepressants (OR = 1.23), and psychotic symptoms (OR = 2.52), increased the risk of TRD (Kautzky et al., 2019). In addition, the European study and our study highlighted that treatment history in an acute ward (inpatient status) and number of past psychiatric admissions are key predictors of TRD. However, more ECT uses were still a reliable factor for TRD. A prospective study, which revealed that among patients with MDD, patients with TRD had more severe depression at baseline, had more past psychiatric admissions, received more augmentation drugs at baseline, received more ECTs in the past, and had longer durations of depressive episodes than treatment responders (Amital et al., 2008).

We further demonstrated that number of psychiatric admissions is an independent factor for predicting treatment resistance in patients with MDD. A study indicated that among patients with MDD, individual depressive levels (as evaluated using depressive ratings) were significantly associated with gender, age, marital status, education, occupation, and number of psychiatric admissions (Shih et al., 2020). However, our results indicated that among the aforementioned factors, only the number of psychiatric admissions predicted treatment resistance (i.e., ECT history, and rTMS treatment outcomes) in patients with MDD who had at least one failed antidepressant trial (Table 2). Furthermore, we discovered that having three or more admissions was significantly associated with an MSM score of ≥10 (17.9%, *p* < 0.05) and a history of rTMS treatment failures (9.4%, *p* < 0.05). We proposed that having three or more psychiatric admissions may be used as a threshold for identifying patients with high treatment resistance (Supplementary Table 1).

Our results also revealed that a diagnosis of OCD is a reliable predictor of treatment resistance (i.e., ECT treatment history) (Table 2). The comorbidity of MDD with other psychiatric disorders has been frequently observed and OCD is one of these comorbidities (Li et al., 2012b). Relative to patients without both MDD and OCD, those with both MDD and OCD exhibit higher levels of symptom severity and respond more poorly to treatment (Fineberg et al., 2005; Quarantini et al., 2011). However, similar to ECT history, OCD is uncommon among patients with MDD, and its clinical value as a predictor is thus limited. In the present study, only eight subjects (3.8%) with MDD had OCD as a comorbidity (Table 1). The prevalence of OCD (as a comorbidity) that was reported in our study was reasonably accurate because a similar prevalence estimation was made using the Nationwide Insurance Database, that is, an increase from 3.6% in the year 2000 to 4.6% in 2013 (Li et al., 2012a). Thus, we proposed the presence of OCD may be incorporated for evaluating levels of TRD (Supplementary Table 1).

We also discovered that life stress level predicted treatment resistance (i.e., ECT treatment history; Table 2). The results were within expectations because our previous study, in which we evaluated the difficulties encountered by people after stressful life events, also indicated that patients with TRD experience higher levels of stress-related psychological distress than patients without TRD (Kimura et al., 2015). Similarly, a study investigated whether stressful life events are an independent risk factor for TRD, and it reported that patients with MDD, those with TRD experienced more stressful life events relative to treatment responders (*n* = 107) (Amital et al., 2008).

This study had some limitations. First, the study only recruited patients who received rTMS or iTBS interventions. These results may not be generalized to the entire MDD population. Second, the present study was a retrospective study and patients with intact records of demographic data, clinical variables, and symptomatic ratings were included. However, since we analyzed data from a center specifically designed for TRD treatment, most of the patients had intact records for these depression-related factors. Further prospective studies are still warranted to confirm the findings. Finally, we only evaluated demographic variables and clinical factors associated with treatment response. Recent research has suggested that combining clinical factors with specific biomarkers (e.g., brain signals, neural activities, and cortical excitability) may further improve the accuracy of predicting MDD treatment outcome (Hopman et al., 2021; Ikawa et al., 2022; Lissemore et al., 2022).

## Conclusion

The current study revealed that, in addition to MSM subitems, several clinical variables (e.g., number of psychiatric admissions, OCD as a comorbidity, and life stress level) were reliable clinical factors associated with higher levels of TRD. Among these clinical variables, number of psychiatric admissions is the most robust factor for predicting rTMS responses and a ECT treatment history. In addition, underutilization of ECT is common and the clinical value of a ECT treatment history is limited because patients with MDD rarely receive ECT.

## Data availability statement

The raw data supporting the conclusions of this article will be made available by the authors, without undue reservation.

## Ethics statement

The studies involving human participants were reviewed and approved by Taipei Veterans General Hospital. As this is a retrospective study, a chart review and a waiver of informed consent were approved by the local IRB.

## Author contributions

C-TL and C-MC conceived of and designed the study. C-TL, T-PS, C-MC, M-HC, Y-MB, and S-JT recruited the patients and performed the experiments. C-TL, C-MC, and M-HC analyzed the data. C-TL wrote the manuscript. All authors read and approved the final version of the manuscript.
