# Supplementary material for: Factors associated with antidepressant responses to repetitive transcranial magnetic stimulation in antidepressant-resistant depression
Source: Front Neurosci. 2022 Dec 2;16:1046920. doi: 10.3389/fnins.2022.1046920 (PMC9757691; doi:10.3389/fnins.2022.1046920)
Supplement: Supplementary file 1 [file Table_1.DOCX]

**Table S1. Comparisons of different staging methods and a proposed TRD severity scale**

|  | Thase & Rush model ^9^ | ESM ^10^ | MGH ^11^ | ATHF-SF ^12^ | MSM ^13^ | Proposed model |
| --- | --- | --- | --- | --- | --- | --- |
| ATD failures | -Stage 0 (S0): any inadequate ATD  -S1: failure of >=1 adequate -ATD  -S2: failure of >=2 classes of ATDs  -S3: S3 +1 failed adequate TCA  -S4: S4 +1 failed adequate MAOI | -TRD: defined as >= 2 failed adequate ATDs from 2 classes | -Failed ATD at adequate dose for at least 6 weeks [1 pSoint for every trial]  -ATD optimization (dose or duration) [0.5 point for every trial]  As evaluated by ATRQ | -Evaluation of every ATD (e.g., SSRI, SNRI, TCA, MAOI, other ATDs…) to be rated as “adequate” or not | -Level 1: 1-2 ATDs [1 point]  -Level 2: 3-4 ATDs [2 points]  -Level 3: 5-6 ATDs [3 points]  -Level 4: 7-10 ATDs [4 points]  -Level 5: >10 ATDs [5 points] | **-Level 0: 1 ATD [1 point]**  -Level 1: 2 ATDs [1 point]  -Level 2: 3-4 ATDs [2 points]  -Level 3: 5-6 ATDs [3 points]  -Level 4: 7-10 ATDs [4 points]  -Level 5: >10 ATDs [5 points] |
| Augmentation or combination | X | X | -Augmentation or combination strategy [0.5 point for every trial] | -Evaluation of every non-ATD, augmentation, combination, and psychotherapy to be rated as “adequate” or not | -Augmentation Used [1 point] | -Augmentation Used [1 point] |
| ECT or other brain stimulations | -Stage 5 (S5): S4+ 1 failed course of bilateral ECT | X | -Failed ECT [3 points] | -Evaluation of ECT, TMS, and VNS to be rated as “adequate” or not | -ECT Used [1 point] | -ECT Used [1 point] |
| Illness Duration | X | -TRD1: 12-16 weeks  -TRD2: 18-24 weeks  -TRD3: 24-32 weeks  -TRD4: 30-40 weeks  -TRD5: 36 weeks-1 year | X | X | -Acute (<=12 months) [1 point]  -Sub-acute (13-24 months) [2 points]  -Chronic (>24 months) [3 points] | -Acute (<=12 months) [1 point]  -Sub-acute (13-24 months) [2 points]  -Chronic (>24 months) [3 points] |
| Symptom Severity | X | X | X | X | -Sub-syndromal [1 point]  -Mild [2 points]  -Moderate [3 points]  -Severe [4 points]  -Severe with psychosis [5 points] | -Sub-syndromal [1 point]  -Mild [2 points]  -Moderate [3 points]  -Severe [4 points]  -Severe with psychosis [5 points] |
| Psychiatric admissions | X | X | X | X | X | -Psychiatric admission x 1 [1 point]  -Psychiatric admissions x >=3 [2 point] |
| OCD comorbidity | X | X | X | X | X | -Comorbid OCD [1 point] |

Note. ESM, European Staging Method; MGH, Massachusetts General Hospital staging model; MSM, Maudsley Staging Methods; ATHF-SF, Antidepressant Treatment History Form-Short Form; ATD(s), antidepressant(s); ATRQ, Antidepressant Treatment Response Questionnaire; ECT, electroconvulsive therapy; APD, antipsychotic(s); TMS, Transcranial Magnetic Stimulation; VNS, Vagus Nerve Stimulation; TCA, tricyclic antidepressant; MAOI, monoamine oxidase inhibitor; NA, not available.

A modified MSM method is proposed that incorporates number of psychiatric admissions and OCD as a comorbidity and defines Level 1 of antidepressant failure as having failed one or two antidepressant trials.
